# Validation of a modified South African triage scale in a high-resource setting: a retrospective cohort study

**DOI:** 10.1186/s13049-023-01076-y

**Published:** 2023-03-20

**Authors:** Dagfinn Lunde Markussen, Heidi Synnøve Brevik, Rune Oskar Bjørneklett, Mette Engan

**Affiliations:** 1 Emergency Care Clinic, Haukeland University Hospital, 5021 Bergen, Norway; 2 Department of Clinical Science, University of Bergen, Postboks 7804, 5020 Bergen, Norway; 3 Department of Paediatric and Adolescent Medicine, Haukeland University Hospital, 5021 Bergen, Norway; 4 Department of Clinical Medicine, University of Bergen, Postboks 7804, 5020 Bergen, Norway

## Abstract

**Background:**

Triage systems are widely used in emergency departments, but are not always validated. The South African Triage Scale (SATS) has mainly been studied in resource-limited settings. The aim of this study was to determine the validity of a modified version of the SATS for the general population of patients admitted to an ED at a tertiary hospital in a high-income country. The secondary objective was to study the triage performance according to age and patient categories.

**Methods:**

We conducted a retrospective cohort study of patients presenting to the Emergency Department of Haukeland University Hospital in Norway during a four-year period. We used short-term mortality, ICU admission, and the need for immediate surgery and other interventions as the primary endpoints.

**Results:**

A total of 162,034 emergency department visits were included in the analysis. The negative predictive value of a low triage level to exclude severe illness was 99.1% (95% confidence interval: 99.0–99.2%). The level of overtriage, defined as the proportion of patients assigned to a high triage level who were not admitted to the hospital, was 4.1% (3.9–4.2%). Receiver operating characteristic (ROC) curves showed an area under the ROC for the detection of severe illness of 0.874 (95% confidence interval: 0.870–0.879) for all patients and 0.856 (0.837–0.875), 0.884 (0.878–0.890) and 0.869 (0.862–0.876) for children, adults and elderly individuals respectively.

**Conclusion:**

We found that the modified SATS had a good sensitivity to identify short-term mortality, ICU admission, and the need for rapid surgery and other interventions. The sensitivity was higher in adults than in children and higher in medical patients than in surgical patients. The over- and undertriage rates were acceptable.

**Supplementary Information:**

The online version contains supplementary material available at 10.1186/s13049-023-01076-y.

## Background

Triage is a process of determining a patient’s priority according to the patient’s illness severity. Ideally, a triage tool has the ability to discriminate between patients with severe illness in need of emergency treatment, and the less ill patients, with high accuracy. Furthermore, a triage tool should be rapid to perform and reliable, with high agreement between observers. Several triage instruments have been developed, and most classify patients into different emergency levels based on symptoms and clinical signs [1]. Triage systems are often developed based on expert opinion and are not always validated [2].

The South African triage scale (SATS) is a noncommercial triage system developed in 2004 for pre-and in-hospital emergency units throughout South Africa. It contains an adult and a paediatric version [3]. In 2012, permission was obtained, and a modified version of the SATS was implemented at the emergency department (ED) at Haukeland University Hospital, Bergen [4]. The English user guide is available on the web page of SATS Norge [5]. After a pilot period from 2012 to 2014, both the SATS-N and available commercial triage system were assessed for implementation in the rest of the Hospital Thrust. The SATS was chosen because it was found easy to use, modifications to local health care settings were allowed, and the favorable economic aspects of a noncommercial triage system. From 2017 the SATS-N was used in all emergency medicine services (EMS) and EDs in the Western Norway Hospital Thrust.

The modified SATS is a five-level triage scale consisting of a clinical discriminator list and a numerical triage early warning score (TEWS). The clinical discriminators contain symptoms, signs, and anamnestic information that require urgent attention even in the absence of abnormal vital signs. The TEWS is based on the measurement of vital parameters organized in age-specific TEWS tables. Each vital sign is assigned a numerical value, and the values are added together to give a total score.

It is essential to determine a triage system’s ability to distinguish between high and low-acuity patients. Triage systems have been shown to perform differently in different age groups and patient groups, and they often have lower performance in children and elderly patients [6–10]. Some studies have also found different performances in medical fields, such as medical and surgical specialties [11, 12].

The correct classification of high-acuity patients concerns patient safety because misclassification of high-acuity patients can cause a delay in diagnosis and treatment, potentially leading to morbidity or mortality. The correct classification of low-acuity patients increases the efficiency of the ED flow and reduces waiting times for truly high-acuity ED visits. Furthermore, the misclassification of low-acuity patients may lead to the overuse of limited recourses. There is no gold standard for classifying true patient urgency. However, short-term mortality and intensive care unit (ICU) admission are often considered reference standards for high urgency, and discharge after ED visits is often considered a reference standard for low urgency [1, 13].

The performance of the original SATS has mainly been studied in resource-limited settings [14–19]. The validity and reliability of the SATS Norway (SATS-N) have not yet been established.

The main objective of this study was to determine the validity of the SATS-N for the general population of patients admitted to an ED at a tertiary hospital in a high-income country. The secondary objective was to study triage performance according to age and patient categories.

## Methods

We conducted a single-centre retrospective cohort study at the main ED at Haukeland University Hospital, Bergen, Norway. Haukeland University Hospital serves a population of approximately 500,000 and functions as a referral hospital for approximately 1,000,000 inhabitants. The yearly ED admission during the study period ranged from 33,000 to 38,000. Triage by the SATS-N is performed on all patients upon arrival. According to hospital guidelines, the triage nurse should assess patients within 15 min of arrival at the ED. The ED handles patients with medical, surgical, and neurologic conditions. Children with medical conditions and females with obstetric conditions are treated elsewhere.

We included all patients who presented to the ED from May 2013 through September 2017. The time interval was defined by the start-up for SATS-N in 2013 and the date for change in database storage system that was carried out in October 2017. To ensure unambiguous patient identification and reliable information regarding mortality, we only included patients with a national identification number. The national identification number is an eleven-digit number that are assigned to all Norwegian citizens at birth, and to all residents upon immigration. It contains embedded information about date of birth and sex.

Data was collected from the electronic journal system used in the ED (Akuttdatabasen, Helse-Vest IKT, version 1.5.5., Stavanger) that contains both administrative data including time of admission, department, source of admission, patient’s national identification number, and clinical data such as age, gender and triage level. Using the national identification number, we linked the data to the Norwegian National Population Register, which contains information about deaths.

Triage nurses categorize patients into triage level 1—red (emergency), 2—orange (very urgent), 3—yellow (urgent), 4—green (not urgent), or 5—blue (can wait). The physician should assess the patient immediately if a patient is triaged into level red, within 10 min when into level orange, within 60 min when into level yellow, and within 120 min when into level green. The triage level blue includes patients arriving for administrative causes or planned visits. Do not resuscitate orders and other treatment limitations are not considered in the triage system. The nurses may not reduce a triage level without consulting a physician.

The reference standard for high-acuity was defined as (1) death within 24 h after presentation to the emergency room, (2) transfer to the ICU from the ED, and/or (3) transfer to the surgical operating theatre (including for coronary angiography) directly from the ED. Mortality at 24 h was collected as well as death within 7 days and 30 days from presentation to the ED. The composite of these outcomes is hereafter referred to as “severe illness”. In addition, mortality within seven days and 30 days after presentation to the ED was considered a proxy for high-acuity at admission. We considered discharge from the ED (i.e., patients not admitted to the hospital) as a reference standard for low acuity.

Categorical data were presented as counts with the percentage of the total. The triage priorities were dichotomized into high (red and orange) or low (yellow, green and blue) triage levels. The association between the triage level and the dichotomous admission level (discharged vs. hospitalized) and other outcomes was evaluated with the help of a cross-classified-table and a χ^2^ test. Odds ratios (OR) for death, admittance to ICU/surgery and severe illness when assigned to the high triage level, and the OR for discharge if assigned to a low triage level, were calculated. The association between severe illness and the five triage levels was assessed by the area under the curve (AUC) of the receiver operating characteristic curve. A two-sided level of statistical significance was set at *p* ≤ 0.05.

Sensitivity was defined as the proportion of patients with severe illness assigned to a high triage level. Specificity was defined as the proportion of patients who did not have severe illness assigned to a low triage level. The positive predictive value (PPV) was defined as the proportion of patients assigned to the high triage levels who had severe illness, and the negative predictive value (NPV) was defined as the proportion of patients assigned to the low triage levels who did not have severe illness. Overtriage was defined as the proportion of patients triaged into the high triage level, who were not hospitalized, and undertriage was defined as the proportion of patients assigned to the low triages level who had severe illness.

The data for sensitivity, specificity, PPV and NPV were calculated with 95% confidence intervals (CI). To study the validity in the subgroups of patients, we also analysed data that were stratified according to medical and surgical conditions and different age groups (children (< 18 years), adults (18–65 years) and elderly (> 65 years). Statistical analyses were performed using IBM SPSS® Statistics (Statistical Package for the Social Sciences) version 26.0 (IBM Corp, Armonk, NY, the United States of America) and GraphPad Prism version 8.0.0 for Windows, GraphPad Software, San Diego, California USA, www.graphpad.com.

## Results

**Fig. 1:**
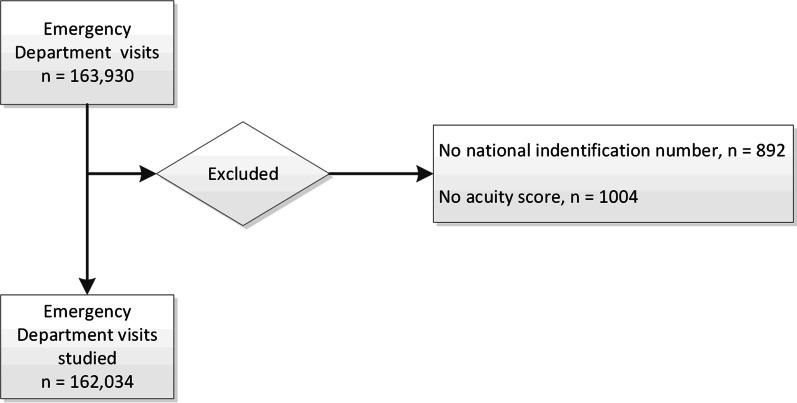
Flow chart of the patients included in the study

We studied data from 163,930 ED presentations in 112,539 patients aged 0–106 years. The patient characteristics, triage level distribution, and health outcomes are presented in Table 1. Due to a short change in the database, admission data from the 17th to the 25th of November 2015 were missing. We do not know the exact number of patients seen in the ED during this period, but based on the mean numbers; this should amount to approximately 900. A total of 1896 ED admissions were excluded from the analysis because of missing data (Fig. 1). Data for time to triage score was available for 73,549 patients. The median time from arrival to triage score recorded was 27.0 min (IQR 15.0–47.0). In total, 96.5% of patients had a triage score registered within 120 min of arrival.

**Table 1:** Patient characteristics and distribution according to triage level

Characteristics	n (%)
Sex	
Females	77,049 (47.6)
Males	84,982 (52.4)
Age groups by years	
< 18	10,214 (6.3)
18–65	83,646 (51.6)
> 65	68,174 (42.1)
Triage level	
1—emergency	16,433 (10.1)
2—very urgent	26,824 (16.6)
3—urgent	59,413 (36.7)
4—not urgent	57,280 (35.4)
5—can wait	2072 (1.3)
Diagnosis	
Medical	93,130 (57.5)
Surgical	68,389 (42.2)
Missing	515 (0.3)
Outcome	
Discharged from ED	29,731 (18.3)
Transfer to ICU or surgery	6932 (4.3)
Severe illness*	7499 (4.5)
24 h mortality	799 (0.5)
7 days mortality	2312 (1.4)
30 days mortality	5393 (3.3)

*ED* emergency department, *ICU* intensive care unit

*Mortality within 24 h after presentation to the emergency room, transfer ICU from the ED and/or transfer to the surgical operation theater (including coronary angiography) directly from the ED

**Fig. 2:**
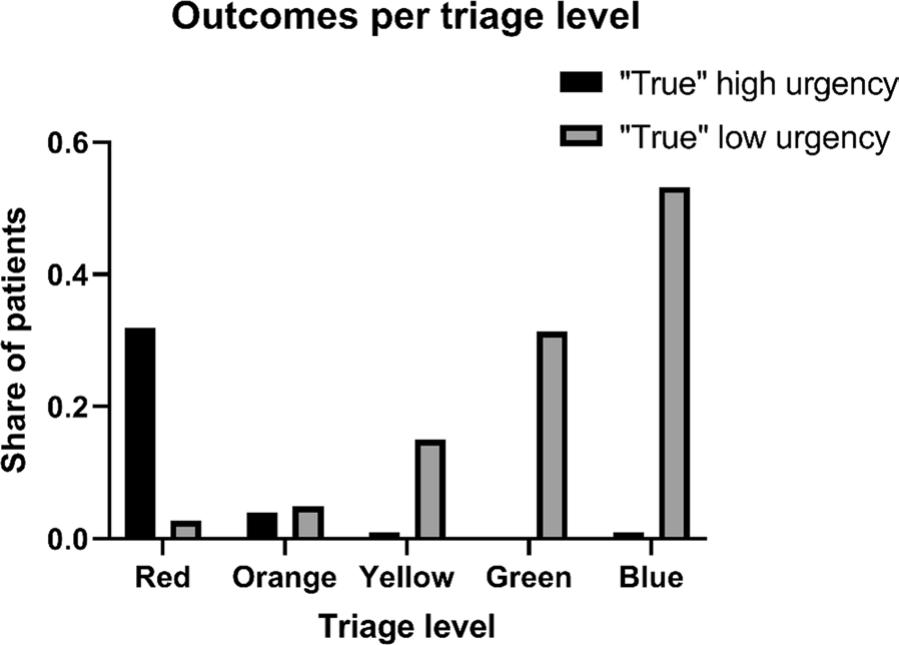
Outcomes per triage level. *True high urgency* was defined as a composite outcome of death within 24 h after presentation to the emergency room, transfer to the ICU from the ED, and/or 3) transfer to the surgical operating theatre (including for coronary angiography) directly from the ED. *True low urgency* was defined as not being admitted from the ED. *Abbreviations ICU* intensive care unit, *ED* emergency department

There were 799 (0.5%), 2312 (1.4%), and 5393 (3.3%) deaths after 24 h, 7 days, and 30 days, respectively. A total of 6932 (4.2%) patients were transferred to the ICU or underwent surgery from the ED, and 7499 (4.6%) patients had the composite outcome of death within 24 h after presentation to the emergency room, transfer to the ICU from the ED, and/or transfer to the operation room (including for coronary angiography) directly from the ED. There were 132,303 (80.2%) ED presentations resulting in further admission to a hospital ward, while 29,731 (18.3%) were discharged from the ED. The admission rate increased with increasing triage level (Fig. 2). Among all the patients admitted to the hospital ward from the ED, 788 of 131,515 (0.6%) died within 24 h, while 11 of 29,720 (0.04%) among those discharged directly from the ED died within 24 h. Elderly patients were more often admitted to a hospital ward than the youngest age group. Among the elderly individuals, 89.3% were admitted to the hospital, while the corresponding proportions in the 18–65 year olds and children were 78.3% and 58.5%, respectively. The proportion of patients who died within 24 h after ED presentation was higher in the oldest age group when than in the other age groups (*p* < 0.0001). The 24-h mortality was 0.9% in the elderly individuals, 0.2% in the adults, and 0.1% in the children. However, the proportion of patients with severe illness, as defined earlier, was lower in the elderly patients than in the other patients (*p* = 0.035). The other endpoints and triage level distribution per age group are available in the Additional file 1: Table. A significantly higher proportion of the surgical patients were discharged directly from the ED when compared to the medical patients, with proportions of admissions of 30.3% and 9.7%, respectively (*p* < 0.0001).

A high triage level was strongly associated with death at 24 h, seven days and 30 days after arrival in the ED (*p* < 0.001). When we compared the patients with high (red and orange) and low (yellow, green and blue) triage levels, we found an ORs (95% CI) of 26.1 (20.7–33.1), 10.1 (9.1–11.1) and 4.7 (4.4–5.0) for 24 h, seven day and 30 day mortality, respectively. For the patients classified to having a low triage level, the 24-h mortality was 0.06% compared to 1.7% in patients with a high triage level (*p* < 0.0001). For transfer to the ICU/surgery and the composite outcome the OR was 18.7 (17.5–20.0) and 19.1 (17.8–20.4), respectively. In the patients given a high triage level, 5923 (13.7%) were transferred to the ICU and/or to the surgical operating theatre (including for coronary angiography) directly from the ED, while the corresponding number for the patients with a low triage level was 1000 (0.8%, *p* < 0.0001). Table 2 shows the outcomes per triage level. The proportion of patients that were undertriaged was 0.90% (95% CI 0.86–0.97%) (Table 3).

**Table 2:** Outcomes per triage level

Triage level	24 h mortality	Admission to ICU/surgery	Not admitted
	n (%)	n (%)	n (%)
1—red (emergency)	641 (3.9%)	4869 (29.6%)	447 (2.7%)
2—orange (very urgent)	81 (0.3%)	1054 (3.9%)	1307 (4.9%)
3—yellow (urgent)	60 (0.10%)	714 (1.2%)	8916 (15.0%)
4—green (not urgent)	15 (0.03%)	266 (0.5%)	17,952 (31.3%)
5—blue (can wait)	2 (0.10%)	20 (1.0%)	1109 (53.2%)

*ICU* intensive care unit

**Table 3:** Sensitivity, specificity, PPV, NPV and the level of overtriage

Patient group	Sensitivity % (95% CI)	Specificity % (95% CI)	PPV % (95% CI)	NPV % (95% CI)	Overtriage % (95% CI)
< 18	69.6 (65.3–73.6)	88.5 (87.8–89.1)	23.1 (20.9–25.3)	98.3 (98.0–98.6)	15.5 (13.7–17.5)
18–65	85.8 (84.6–86.8)	78.4 (78.1–78.7)	16.4 (15.9–16.9)	99.1 (99.0–99.2)	5.1 (4.8–5.4)
> 65	88.0 (86.8–89.2)	71.6 (71.3–72.0)	12.8 (12.3–13.2)	99.2 (99.1–99.3)	2.3 (1.7–3.0)
Medical	91.3 (90.4–92.1)	68.6 (68.3–68.9)	12.9 (12.5–13.3)	99.4 (99.3–99.4)	2.3 (1.8–2.8)
Surgical	77.1 (75.5–78.6)	86.2 (86.0–86.5)	20.3 (19.5–21.0)	98.8 (98.7–98.9)	9.1 (8.6–9.7)
All patients	85.7 (84.8–86.4)	76.2 (76.0–76.4)	14.9 (14.5–15.2)	99.1 (99.0–99.2)	4.1 (3.9–4.2)

Sensitivity—proportion of patients who have the composite outcome (1) death within 24 h after presentation to the emergency room, (2) transfer to Intensive Care Unit (ICU) from the ED and/or (3) transfer to the operation room (including coronary angiography) directly from the ED) assigned to high triage levels

Specificity—the proportion of patients who did NOT have the composite outcome that were assigned to low triage levels

PPV- the proportion of patients assigned to high triage levels who have the composite outcome

NPV—the proportion of patients assigned to low triage levels who do not have the composite outcome

Overtriage—the proportion of patients assigned to high triage levels who were discharged from the ED

*CI* confidence interval, *PPV* positive predictive value, *NPV* negative predictive value

**Fig. 3:**
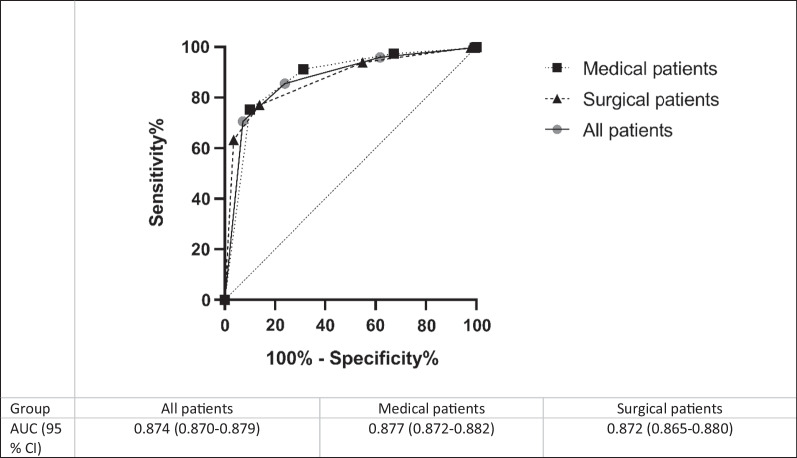
Receiver operating characteristic (ROC) curves and associated area under the ROC (AUROC) for the detection of severe illness for all the patients, the medical and surgical patients. *Abbreviations AUC* area under the curve, *CI* confidence interval

**Fig. 4:**
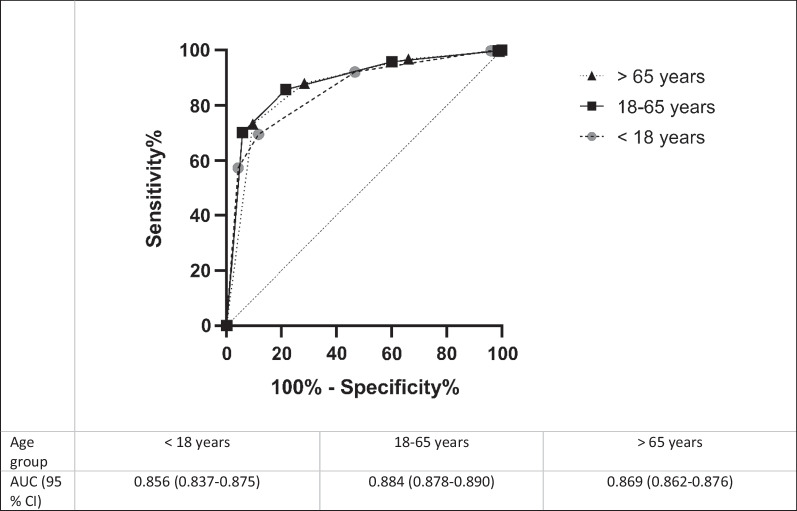
Receiver operating characteristic (ROC) curves and associated area under the ROC (AUROC) for the detection of severe illness for different age groups. *Abbreviations AUC* area under the curve, *CI* confidence interval

The sensitivity, specificity, PPV, and NPV for severe illness, including stratified data for age groups and patient categories, are presented in Table 3. For all the patients, we found an area under the curve (AUC) of 0.874 (Fig. 3). We also analysed the data for the medical and surgical patients separately, and the composite endpoint was strongly associated with high triage levels for all groups. Data for discipline were missing for 515 patients. For all age groups, there was a significant difference in all the outcomes in the patients with a high triage level. The AUCs were 0.586, 0.884 and 0.869 for the children, the adults and the elderly patients, respectively (Fig. 4). The percentage of patients with high triage levels was higher with increasing age. A total of 31.1% of the elderly patients were given a high triage level compared to 23.5% in other age groups (*p* < 0.0001).

A total of 29,731 (18.3%) patients were discharged from the ED. More children (41.5%) and fewer elderly (10.7%) patients were discharged from the ED than adults (21.7%), *p* < 0.01). A low triage level was significantly more frequent in the patients who were not admitted to the hospital (23.6 versus 4.1%, *p* < 0.001). Figure 2 describes the proportion of patients not admitted to the hospital per triage level.

We found an OR (95% CI) of 7.3 (6.9–7.6) for not being admitted if assigned to a low triage level. A higher proportion of the surgical patients were discharged from the ED than the medical patients with proportions of discharge of 30.3% and 9.7%, respectively (*p* < 0.001). The level of overtriage is shown in Table 3.

## Discussion

In this retrospective study, we found a strong association between mortality, transfer to the ICU from the ED, and/or transfer to the operation room, and high triage level with the modified SATS. As expected, the triage level was more strongly associated with 24-h mortality than with 30 day mortality. The sensitivity and specificity to detect severe illness were 85.7% and 76.2%, respectively. Overtriage was relatively common, especially in children and surgical patients, while undertriage was uncommon (0.9%). This is, to our knowledge, the first study to assess the validity of the SATS in adults in a high-resource setting.

We found that the NPV, i.e., the ability of a low triage level to exclude a serious outcome was excellent and above 98% in all subgroups. However, this comes at the cost of a large proportion of patients assigned to high triage levels, who did not have the outcomes measured in this study. This does not necessarily mean that all these patients were overtriaged. Some may have needed urgent interventions that were not recorded in this study such as fibrinolytic therapy for ischemic stroke, antimicrobial therapy for serious infections not requiring ICU admission, and acute interventions not performed in the operating room such as vascular interventions performed in the Radiology Department. Furthermore, undertriage is a major concern as it can lead to delayed or inadequate treatment, which can result in poor outcomes and even death. When patients are not triaged properly and are not given the appropriate level of care in a timely manner, it can lead to a deterioration of their condition and a greater likelihood of complications. On the other hand, overtriage can lead to unnecessary testing and treatment, increased healthcare costs and longer wait times, but it does not put the patient's health at the same risk as undertriage. Compared to the findings in other studies, our study shows that the SATS-N performs as well as or better than other triage systems used in high-resource settings [20–22]. A systematic review found that most studies of emergency department triage systems reported a low sensitivity (< 80%) in identifying patients who had critical illness outcomes or died during hospitalization [1]. In a recent systematic review of triage systems in emergency care, the authors found that common triage systems had a sensitivity for ICU admission ranging from 58 to 88% in adults and 71–93% in children. The specificity of triage systems to accurately classify patients discharged home as low urgency ranged from 64 to 98% in adults and 69–96% in children [13]. It is, however, difficult to compare the validity of triage systems between different studies, as the definitions of over and undertriage are very heterogeneous [23]. Other studies of SATS, that were performed in low-resource settings have found a sensitivity from 91 to 96%. However, the studies used hospital admission as the gold standard for high acuity, and the reported mortality in the studies is very low [15, 18, 24].

All validation studies on triage systems are also subject to limitations due to the absence of a gold standard of urgency [23, 25]. We also have very limited knowledge about the effect of triage levels on clinical outcomes. Relevant clinical outcomes could span from patient satisfaction, resource utilization, patient harm due to delay in or improved health due to prompt treatment and assessment, to mortality. No single outcome can measure all of these factors. To validate triage systems it is necessary to construct the best proxy for the reference standard applicable to the whole spectrum of patients in the ED [2]. We chose 24 h mortality, admittance to the ICU or direct transfer to asurgical operating theatre (including for coronary angiography) as a proxy for true high acuity. Not all patients with true high acuity may meet these criteria. Hence, our strict definition of true high acuity might have excluded patients with severe conditions. Regarding true low acuity, which in this study was defined as discharge from the ED, we did not collect data on readmission within a short period of time after discharge. Hence, some patients might have been misclassified as low acuity patients, even though their clinical condition indicated more severe illness.

This study has some limitations concerning generalizability. First, the hospitalization rate in our hospital system is higher than that in many countries. In other studies on triage performance, the hospitalization rate ranges from 4 to 58% [13]. In Norway, the organization of the emergency health care system differs from many other countries in that it is based on a two-tiered system where the municipalities are responsible for the first part, and the hospital trusts are responsible for the more specialized part of the system. In a study of Norwegian emergency admissions, Blinkenberg and collaborators found that a out-of-hospital physician referred 57% of patients presenting to emergency departments were referred by an out-of hospital physician and 43% of patients presented directly, mainly by Emergency medical services [26]. Therefore, most patients who arrive in the ED are admitted to the hospital. Indeed, in our study, 81.7% of the patients were hospitalized. In this study, the findings for true low-urgency patients may therefore not be generalizable to other health systems with higher discharge rates directly from the ED.

Second, our ED only takes care of children with surgical conditions, and our findings may not be applied to children with medical conditions. However, we have previously published a study on the modified SATS in children with medical conditions, and a similar sensitivity was found in that study [9]. This was also a single centre study, and the performance of SATS-N may be different in other EDs.

A strength of our study is the use of data from the National Population Register to ensure that no deaths were missed. Mortality is a definite endpoint, and the ideal triage system would classify those with a high mortality risk into the highest acuity levels. Most studies that have looked at mortality in relation to triage level have used either ED mortality or in-hospital mortality as the endpoint [13]. The disadvantage of using these endpoints is that deaths that occur shortly after discharge are missed. Indeed, in our study, 11 patients discharged from the ED died within 24 h after ED presentation, and 76 and 220 of the patients who were not admitted died after 7 and 30 days respectively. Another strength of our study is that our definitions and assessment of triage performance are based on objective patient outcome markers and not on subjective expert opinions, as is the case in many studies on triage performance [23, 27–30].

In this singe centre study, we validated the SATS-N for a few large groups of patients in the ED. Future multicentre studies, preferably including other emergency settings, should expand on the results from this study to further establish the validity of the SATS-N. Moreover, effort should be made to identify possible smaller subgroups that may be at risk of undertriage by the triage system.

## Conclusions

In summary, we found that the modified SATS had excellent sensitivity to identify short-term mortality, ICU admission, and the need for immediate surgery and other interventions. The sensitivity was higher in adults than in children and higher in medical patients than in surgical patients. The over- and undertriage rates were acceptable.

## Supplementary Information

**Additional file 1.** Outcomes per Triage Level for Different Age Groups. This file contains data on the outcomes (24 hour mortality, admission to ICU/surgery and discharege from the ED) for patients, grouped by triage level and age group. The data is presented in table format and provides a detailed analysis of the impact of triage level on outcomes for different age groups.

## Data Availability

The datasets used and/or analysed during the current study are available from the authors on reasonable request and in compliance with the General data protection regulation and Norwegian legislation.

## Abbreviations

SATS: South African Triage Scale
ROC: Receiver operating characteristic
ED: Emergency Department
TEWS: Triage early warning score
ICU: Intensive care unit
SATS-N: SATS Norway
OR: Odds ratio
PPV: Positive predictive value
NPV: Negative predictive values
CI: Confidence interval
AUC: Area under the curve
