# Supplementary material for: Validation of a modified South African triage scale in a high-resource setting: a retrospective cohort study
Source: Scand J Trauma Resusc Emerg Med. 2023 Mar 20;31:13. doi: 10.1186/s13049-023-01076-y (PMC10026449; doi:10.1186/s13049-023-01076-y)
Supplement: Supplementary file 1 — Additional file 1. Outcomes per Triage Level for Different Age Groups. This file contains data on the outcomes (24 hour mortality, admission to ICU/surgery and discharege from the ED) for patients, grouped by triage level and age group. The data is presented in table format and provides a detailed analysis of the impact of triage level on outcomes for different age groups. [file 13049_2023_1076_MOESM1_ESM.docx]

Supplementory table - Outcomes per triage level for different age groups

<18 years

| Triage level | 24 h mortality | Admission to ICU/surgery | Not admitted |
| --- | --- | --- | --- |
|  | n (%) | n (%) | n (%) |
| 1- red (emergency) | 8 (1.2 %) | 270 (39.9 %) | 114 (16.8 %) |
| 2 - orange (very urgent) | 0 | 59 (7.6 %) | 112 (14.4 %) |
| 3 - yellow (urgent) | 0 | 109 (3.1 %) | 1167 (14.4 %) |
| 4 - green (not urgent) | 0 | 37 (0.8 %) | 2575 (53.3 %) |
| 5 - blue (can wait) | 0 | 1 (0.3 %) | 267 68.6 %) |

16 to 65 years

| Triage level | 24 h mortality | Admission to ICU/surgery | Not admitted |
| --- | --- | --- | --- |
|  | n (%) | n (%) | n (%) |
| 1- red (emergency) | 155 (2.1 %) | 2693 (36.6 %) | 239 (3.3 %) |
| 2 - orange (very urgent) | 11 (0.1 %) | 607 (4.6 %) | 811 (6.1 %) |
| 3 - yellow (urgent) | 10 (0.03 %) | 387 (1.2 %) | 5627 (18.1 %) |
| 4 - green (not urgent) | 3 (0.01 %) | 154 (0.5 %) | 10823 (35.1 %) |
| 5 - blue (can wait) | 1 (0.1 %) | 8 (0.7 %) | 673 (59.4 %) |

>65 years

| Triage level | 24 h mortality | Admission to ICU/surgery | Not admitted |
| --- | --- | --- | --- |
|  | n (%) | n (%) | n (%) |
| 1- red (emergency) | 478 (5.7 %) | 1906 (22.7 %) | 94 (1.1 %) |
| 2 - orange (very urgent) | 70 (0.5 %) | 388 (3.0 %) | 384 (3.0 %) |
| 3 - yellow (urgent) | 50 (0.2 %) | 218 (0.9 %) | 2122 (8.5 %) |
| 4 - green (not urgent) | 12 (0.1 %) | 75 (0.3 %) | 4554 (21.1 %) |
| 5 - blue (can wait) | 1 (0.2 %) | 11 (2.0 %) | 169 (30.1 %) |

Abbreviations: ICU, intensive care unit
